# Brain Structure of South African Children Born to Mothers on Dolutegravir Versus Efavirenz-Based Antiretroviral Therapy

**DOI:** 10.1093/jpids/piag022

**Published:** 2026-03-27

**Authors:** Layla E Bradford, Catherine J Wedderburn, Thokozile R Malaba, Helene Theunissen, Jessica E Ringshaw, Niall J Bourke, Steve C R Williams, Nengjie He, Lucy Read, Catriona Waitt, Helen Reynolds, Angela Colbers, Jim Read, Lauren Davel, Catherine Orrell, Miriam Taegtmeyer, Duolao Wang, Saye Khoo, Landon Myer, Kirsten A Donald

**Affiliations:** Department of Paediatrics and Child Health, University of Cape Town, Cape Town, South Africa; Neuroscience Institute, University of Cape Town, Cape Town, South Africa; Department of Paediatrics and Child Health, University of Cape Town, Cape Town, South Africa; Neuroscience Institute, University of Cape Town, Cape Town, South Africa; Division of Epidemiology & Biostatistics, School of Public Health, University of Cape Town, Cape Town, South Africa; Division of Epidemiology & Biostatistics, School of Public Health, University of Cape Town, Cape Town, South Africa; Department of Paediatrics and Child Health, University of Cape Town, Cape Town, South Africa; Neuroscience Institute, University of Cape Town, Cape Town, South Africa; Department of Neuroimaging, Institute of Psychiatry, Psychology & Neuroscience, King’s College London, London, United Kingdom; Department of Neuroimaging, Institute of Psychiatry, Psychology & Neuroscience, King’s College London, London, United Kingdom; Department of Neuroimaging, Institute of Psychiatry, Psychology & Neuroscience, King’s College London, London, United Kingdom; Global Health Trials Unit, Liverpool School of Tropical Medicine, Liverpool, United Kingdom; Global Health Trials Unit, Liverpool School of Tropical Medicine, Liverpool, United Kingdom; Infectious Diseases Institute, Makerere University, Kampala, Uganda; Department of Pharmacology and Therapeutics, University of Liverpool, Liverpool, United Kingdom; Department of Pharmacology and Therapeutics, University of Liverpool, Liverpool, United Kingdom; Department of Pharmacy, Radboud University Medical Centre, Nijmegen, Netherlands; Global Health Trials Unit, Liverpool School of Tropical Medicine, Liverpool, United Kingdom; Department of Paediatrics and Child Health, University of Cape Town, Cape Town, South Africa; Desmond Tutu HIV Centre, Institute of Infectious Disease and Molecular Medicine, University of Cape Town, Cape Town, South Africa; Department of Medicine, University of Cape Town, Cape Town, South Africa; Department of Clinical Sciences, Liverpool School of Tropical Medicine, Liverpool, United Kingdom; Department of Clinical Sciences, Liverpool School of Tropical Medicine, Liverpool, United Kingdom; Department of Pharmacology and Therapeutics, University of Liverpool, Liverpool, United Kingdom; Division of Epidemiology & Biostatistics, School of Public Health, University of Cape Town, Cape Town, South Africa; Department of Paediatrics and Child Health, University of Cape Town, Cape Town, South Africa; Neuroscience Institute, University of Cape Town, Cape Town, South Africa

**Keywords:** dolutegravir, ART exposure, children, HIV exposure, MRI

## Abstract

**Background:**

The impact of in utero exposure to specific antiretroviral therapy (ART), particularly integrase strand transfer inhibitors, on early brain development, remains poorly understood. We used magnetic resonance imaging (MRI) to compare brain structure in children who are HIV-exposed and uninfected (CHEU) with prenatal dolutegravir (DTG) vs efavirenz (EFV) exposure.

**Methods:**

DolPHIN-2 was a randomized trial of pregnant women initiating DTG- vs EFV-based ART in the third trimester. At 3-4 years of age, a subgroup of their children from the South African cohort, along with HIV-unexposed (CHU) children, underwent T1-weighted MRI (DolPHIN-2 PLUS). Measurements of brain structure, including volume, cortical thickness, and surface area, were extracted using Freesurfer. Associations between ART /HIV exposure and child brain structure were examined using multiple linear regression.

**Results:**

This analysis included 58 children (25 CHEU [13 DTG, 12 EFV] and 33 CHU, mean age 46.35 months, 51.7% male). Demographic characteristics were similar across groups. Significant differences in global or regional brain volumes, cortical thickness, or surface area were not detected between DTG- and EFV-exposed children or between CHEU and CHU.

**Conclusion:**

Among this small sample of children at 3-4 years, statistically significant differences in global or regional brain structure were not detected between those exposed in the third trimester of pregnancy to DTG or EFV and CHU. Given the modest sample size, the study had limited power to detect small-to-moderate differences. Further longitudinal studies with larger sample sizes are needed to assess the effects of specific ART in the context of new regimens and better maternal HIV disease control on CHEU brain structure.

## INTRODUCTION

There is a growing population of children who are HIV-exposed and uninfected (CHEU), particularly in Sub-Saharan Africa.^1,2^ CHEU are considered a vulnerable population, with evidence indicating an increased risk for adverse health outcomes, including immune, growth, and developmental alterations.^3–7^ Neurodevelopmental differences, including poorer language and motor outcomes, have been noted in CHEU compared to children who are HIV-unexposed (CHU) from similar contexts.^8–10^ This highlights a significant health burden and barrier to realizing the full developmental potential in this population.

One potential contributor to these neurodevelopmental differences is prenatal exposure to antiretroviral therapy (ART). While maternal ART usage during pregnancy is essential for preventing vertical HIV transmission and supporting maternal immune health, the potential neurotoxic effects on fetal brain development remain poorly understood.^11^ There is some evidence suggesting an association between efavirenz (EFV)-based regimens and microcephaly as well as poorer neurodevelopmental outcomes, although findings are inconsistent.^9,10,12–15^ Early life represents a critical and sensitive period of brain growth, and this lack of data limits our ability to fully evaluate the long-term risks and benefits of current ART regimens used during pregnancy.

Neuroimaging, particularly magnetic resonance imaging (MRI), has the potential to offer valuable insight into the neurobiological mechanisms and contributing factors involved in the neurodevelopment of CHEU.^16^ Neuroimaging studies of HIV-exposed but uninfected (HEU) neonates and young children exposed in utero to EFV-based ART have reported alterations in subcortical gray matter regions, including reduced caudate and putamen volumes in early life, as well as changes in white matter microstructure.^17–20^ Notably, a South African neuroimaging study of HEU neonates exposed to EFV-based ART found that those born to mothers who initiated ART before conception had larger caudate volumes than those whose mothers began treatment later in pregnancy, suggesting that longer ART duration may protect against subcortical brain alterations in CHEU.^17^ This suggests ART, by improving maternal immune functioning through viral suppression, may offer neuroprotection to the developing fetus. Consistent with these findings, other studies have found associations between poor maternal immune status (low maternal CD4 counts and high viral loads) and smaller subcortical brain volumes, reinforcing the connection between maternal immune regulation and fetal brain development in the context of HIV.^18,19^ Importantly, reported EFV-associated brain differences appear more prominent in neonates and infancy, with less consistent findings reported in older childhood, indicating that early EFV-related brain differences may be transient or developmentally moderated.

While there is a global effort to develop safer and more efficacious drugs for the treatment of HIV, pregnant women are often excluded from ART clinical trials due to safety concerns, resulting in limited data on the safety and long-term effects of in utero ART exposure. The DolPHIN-2 trial investigated dolutegravir (DTG), an integrase strand transfer inhibitor, in comparison to EFV, a non-nucleoside reverse transcriptase inhibitor, in pregnant women initiating ART in the third trimester.^21^ Results showed that DTG achieved a more rapid and greater viral load suppression by the time of delivery compared to EFV.^21^ However, no studies to date have examined the impact of prenatal DTG exposure on child brain structure.

In this study, we address this gap by comparing brain structure at 3-4 years of age in children exposed in utero to DTG versus EFV, using MRI. Secondary objectives included comparing CHEU and CHU brain structure and examining associations between maternal HIV disease severity and child brain development.

## METHODS

### Participants

DolPHIN-2 PLUS is an observational infant-follow up sub-study of the DolPHIN-2 trial (NCT03249181) in pregnant women who initiated ART (DTG vs EFV) in the third trimester.^21^ The parent trial’s South African site included participants who were recruited from the Gugulethu Midwife Obstetrics Unit, a primary prenatal healthcare facility located in Gugulethu, an informal peri-urban settlement within the Cape Town metropole with a predominantly isiXhosa-speaking population. Gugulethu, like many other informal settlements in South Africa, is impacted by a significant health burden caused by a high prevalence HIV. CHEU eligible for the DolPHIN-2 PLUS study neuroimaging sub-study were identified from the South African DolPHIN-2 cohort and were invited to participate when they reached the age of 3-4.5 years. Invitations were based on feasibility and contactability, and no selection was based on child health status, developmental outcomes, or prior clinical findings from the parent trial. In addition to CHEU, a comparison group of CHU was recruited postnatally from the same geographical area using convenience sampling and were similar in age, sex, and sociodemographic background. Overall, 58 children (25 CHEU and 33 CHU), born between 2018 and 2019, were included in this analysis, aged between 39 and 54 months. Children were excluded if they had a known positive HIV test or MRI contraindication. Written informed consent was obtained from all caregivers prior to participation. The consent process included a detailed explanation of the purpose of the study, MRI procedures, and the potential risks and benefits of MRI scanning in young children. To assess the representativeness of the CHEU neuroimaging sub-sample, maternal sociodemographic and clinical characteristics were compared with those of the broader South African DolPHIN-2 trial cohort.

### Procedures

#### HIV/ART Data

Study procedures in pregnant mothers from DolPHIN-2 have been described previously.^21^ In the third trimester, mothers in the DTG group received dolutegravir (50 mg) plus generic tenofovir disoproxil fumarate (300 mg) co-formulated with emtricitabine (200 mg), and mothers in the EFV group received a generic single fixed-combination pill of efavirenz (600 mg) with tenofovir and emtricitabine. In keeping with national guidelines in South Africa, all newborn infants were prescribed nevirapine for 6 weeks. Maternal viral load was measured at enrolment during the third trimester as well as within 14 days post-delivery. CD4 was measured at enrolment.

#### Sociodemographic Variables

Sociodemographic data for mothers of both CHEU and CHU were collected at the time of the DolPHIN-2 PLUS study visit, when children were aged 3-4 years. Questionnaires included retrospective reporting of pregnancy-related exposures, including the Alcohol, Smoking and Substance Involvement Screening Test (ASSIST). Maternal depressive symptoms were assessed using Edinburgh Postnatal Depression Scale (EPDS). Maternal health data related to HIV infection were only available for mothers enrolled in DolPHIN-2.

#### Neuroimaging Acquisition

Magnetic resonance imaging was conducted during non-sedated natural sleep using a Siemens 3 T Skyra system, housed at The Cape Universities Brain Imaging Centre, located in Cape Town, South Africa. T1-weighted (T1w) MEMPRAGE scans were conducted to obtain brain morphological measures, including cortical thickness, volume, and surface area. The center provided a child-friendly environment for pediatric neuroimaging, and the research team was familiar with the imaging procedures and followed a protocol described previously.^16^

#### Neuroimaging Processing

**Figure 1:**
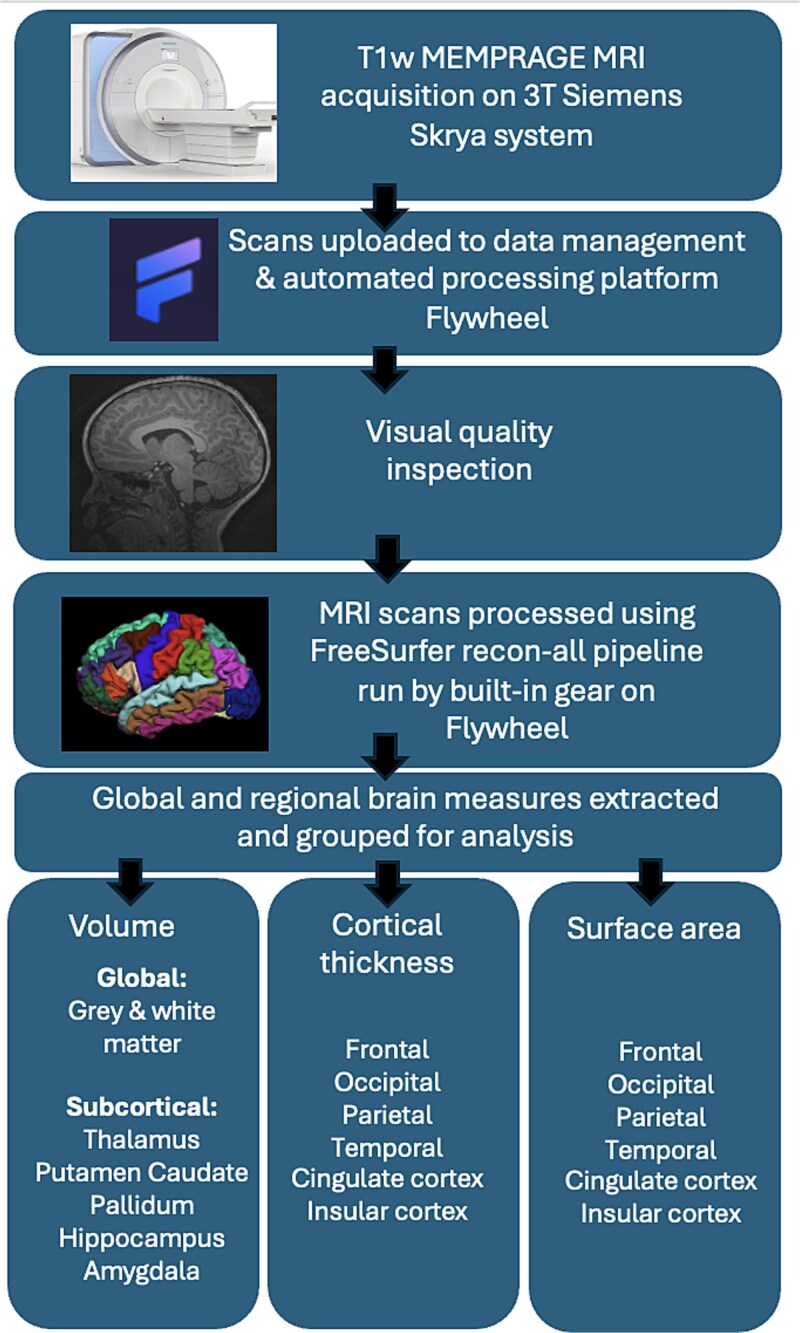
Workflow for Acquisition, Processing, and Analysis of Structural MRI Data in the DolPHIN-2 PLUS Neuroimaging Sub-Study

All MRI data were processed using a standardized automated pipeline, and visual quality inspection was conducted by trained researchers using consistent criteria to assess for the presence of motion artifact. Image processing and analysis were conducted blinded to HIV and ART exposure status. T1w images were processed using Freesurfer version 7.2.0.^22,23^ Cortical reconstruction and volumetric segmentation were performed using the automated Freesurfer pipeline (recon-all command), which was run via an inbuilt gear on Flywheel (https://flywheel.io), a medical imaging and AI platform. As an end-to-end tool for data management, curation, and computational analysis, Flywheel served as the host for centralized image processing. Global and regional subcortical brain volumes, cortical thickness, and surface area were extracted for analysis. This was computed according to the Desikan-Killiany atlas for cortical parcellation and an inbuilt probabilistic atlas for segmentation.^24,25^ Based on prior research on HIV exposure and child brain outcomes, the regions of interest (ROIs) for this study were selected a priori and included total gray matter, total white matter, and several subcortical gray matter structures (thalamus, caudate, putamen, pallidum, hippocampus, and amygdala).^6,17–19^ An overview of the workflow for acquisition, processing, and analysis of structural MRI data is described in Figure 1.

### Statistical Analysis

Sociodemographic and clinical characteristics were compared between groups (CHEU vs CHU and DTG vs EFV). To assess potential selection bias of CHEU, maternal sociodemographic and clinical characteristics of South African DolPHIN-2 PLUS neuroimaging sub-sample were compared with those of the broader South African DolPHIN-2 trial cohort. For normally distributed continuous variables (eg, age at scan, intracranial volume, and maternal mean viral load [log-transformed]), comparisons were conducted using independent samples *t*-tests. The Mann−Whitney *U* test was applied to non-normally distributed continuous variables. Categorical variables were analyzed using chi-squared and Fisher’s exact tests, as appropriate.

Global and subcortical brain regions were selected a priori based on previously reported associations in CHEU. Given the limited existing literature on the effects of specific ART regimens on brain development, an exploratory approach was adopted for cortical thickness and surface area. Associations between exposure status (HIV and ART exposure) with brain structure (brain volume, cortical thickness, and surface area) were examined using multiple linear regression models. To reduce the number of comparisons, bilateral subcortical ROI volumes were averaged. For cortical thickness and surface area, regions were grouped anatomically according to the Desikan-Killiany atlas into frontal, temporal, parietal, occipital, cingulate, and insular lobes.^25^ All models were adjusted for total intracranial volume, sex, and age at scan. Analyses comparing DTG and EFV exposure additionally adjusted for maternal CD4 count.

In secondary analyses, associations between maternal HIV disease severity and child brain morphology were examined. Maternal viral load (measured during pregnancy and at delivery) and CD4 (measured during pregnancy) were evaluated as predictors of brain volume using linear regression. Viral suppression was defined as HIV RNA <50 copies/mL, consistent with the DolPHIN-2 ART trial, reflecting the lower limit of detection of standard clinical assays. Cohen’s *d* effect size was estimated to assess the exposure and treatment effects and categorized according to conventional benchmarks.^26^ Model residuals were assessed graphically to confirm assumptions of normality. All analyses were performed in STATA version 18.0 (StataCorp, College Station, TX), using a two-sided significance threshold of *P* < .05.

## RESULTS

A total of 60 children successfully underwent T1w MRI scans, of which 2 were excluded due to the presence of severe motion artifact on visual quality inspection. The final sample included 58 children (30 male, 28 female) with high-resolution T1w images. Of these, 25 were CHEU (13 male, 12 female, mean age 45.5 months) and 33 were CHU (17 male, 16 female, mean age 47 months). Within the CHEU group, 13 children were DTG-exposed (7 male, 6 female) and 12 were EFV-exposed (6 male, 6 female).

As shown in Table 1, demographic characteristics, including age at scan, sex, maternal education, maternal employment, maternal substance use during pregnancy, and maternal depression, were similar between DTG- and EFV- exposed groups, and between CHEU and CHU. Maternal CD4 count at enrolment (prior to ART initiation) was significantly higher in the DTG group (median 520 cells/mm^3^, IQR 427-797 cells/mm^3^) compared to the EFV group (median 358.5 cells/mm^3^, IQR 289-447.5 cells/mm^3^, *P* = .039). Maternal viral load at enrolment and at delivery did not differ significantly between treatment groups (*P* = .480 and *P* = .378, respectively). At delivery, 11 (84.6%) mothers in the DTG group and 8 (66.7%) in the EFV group were virally suppressed (viral load <50 copies/mL). When comparing maternal sociodemographic and clinical characteristics of the DolPHIN-2 PLUS neuroimaging CHEU sub-sample with those of the broader South African DolPHIN-2 cohort, no statistically significant differences were observed with respect to maternal education, employment status, CD4 count at enrolment, or viral load at enrolment (Table S1). Consistent with the parent trial findings, a higher proportion of mothers in the DTG group achieved viral suppression at delivery compared with the EFV group in both cohorts, although this difference reached statistical significance only in the parent trial (*P* < .001). In the broader South African DolPHIN-2 cohort, maternal depressive symptoms were more frequently reported in the DTG group than in the EFV group; however, no statistically significant difference in depressive symptoms between ART exposure groups was observed in the DolPHIN-2 PLUS neuroimaging sub-sample.

**Table 1:** Socio-Demographic and Clinical Characteristics of Children According to HIV Exposure (CHU vs CHEU) and ART Exposure (DTG vs EFV) Status

Variable	Total (*n* = 58)	CHEU (*n* = 25)	CHU (*n* = 33)	*P*-value	DTG (*n* = 13)	EFV (*n* = 12)	*P*-value
*Sociodemographic characteristics*							
Child age at scan Mean (SD) (months)	46.35 (3.97)	45.50 (3.16)	47.00 (4.42)	.133	45.69 (3.86)	45.25 (2.34)	.730
Sex Male Female	30 (51.7%) 28 (48.3%)	13 (52.0%) 12 (48.0%)	17 (51.5%) 16 (48.5%)	.971	7 (53.8%) 6 (46.2%)	6 (50%) 6 (50%)	.848
Maternal education (completed secondary school)	23 (39.7%)	9 (36.0%)	14 (42.4%)	.620	3 (23.1%)	6 (50%)	.226
Maternal employment status (employed)	21 (36.2%)	12 (48.0%)	9 (27.3%)	.104	5 (38.5%)	7 (58.3%)	.320
Maternal alcohol use during pregnancy	19 (32.8%)	8 (32.0%)	11 (33.3%)	.915	2 (15.4%)	6 (50%)	.097
Maternal depression	8 (13.8%)	2 (8.0%)	6 (18.2%)	.445	1 (7.7%)	1 (8.3%)	1.000
*Neuroanatomical variables*							
Total intracranial volume (cm^3^)	1291.97 (154.23)	1286.62 (162.04)	1296.02 (150.46)	.822	1279.42 (204.08)	1294.42 (108.28)	.819
*Maternal HIV variables in pregnancy*							
Maternal CD4 count, median (IQR) (cells/mm^3^)		440 (346.0-660.0)			520 (427.0-797.0)	358.5 (289.0 -447.5)	.039
≤500 cells/mm^3^		15 (60%)			5 (38.5%)	10 (83.3%)	.041^a^
>500 cells/mm^3^		10 (40%)			8 (61.5%)	2 (16.7%)	
Maternal viral load at enrollment (copies/mL)							
<50 copies/mL		2 (8%)			2 (15.4%)	0 (0%)	.480
≥50 copies/mL		23 (92%)			11 (84.6%)	12 (100%)	
Maternal viral load at delivery (copies/mL)							
<50 copies/mL		19 (76%)			11 (84.6%)	8 (66.7%)	.378
≥50 copies/mL		6 (24%)			2 (15.4%)	4 (33.3%)	

Data are *N* (%), mean (SD), or median (IQR). Continuous variables were compared with unpaired *t*-tests if normally distributed or Mann−Whitney *U* tests if distribution was not normal; categorical variables were compared with chi-squared tests.

^a^ *P* < .05. Abbreviations: CHEU, children who are HIV-exposed uninfected; CHU, children who are HIV-unexposed; DTG, dolutegravir; EFV, efavirenz.

### ART Exposure and Brain Structure

**Figure 2:**
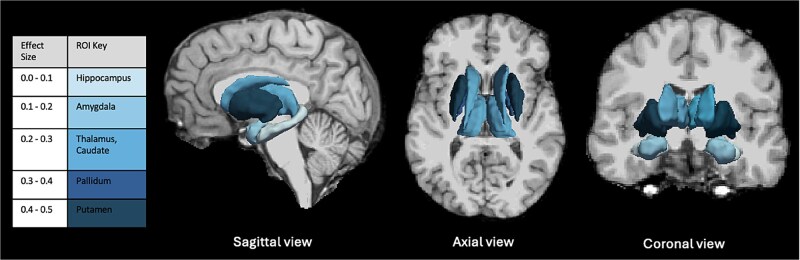
Schematic of the Effect Sizes of Subcortical Brain Volume Differences in CHEU Exposed to DTG vs EFV. The key represents the color coding of absolute effect sizes corresponding to brain volumes in subcortical regions of interest. Schematic created using Freesurfer version 7.2.0. Abbreviations: CHEU, children who are HIV-exposed uninfected; DTG, dolutegravir; EFV, efavirenz; ROI, region of interest.

Total intracranial volume was similar between those children exposed to DTG and EFV (1279 vs 1294 cm^3^, *P* = .819) (Table 1). Within the CHEU group, there were no detectable differences in the brain volumes between DTG- and EFV- exposed children. This included total gray matter (adjusted difference -1386.54 mm^3^, Cohen’s *d* = -0.08 [95% CI -0.86 to 0.71], *P* = .861) and white matter volume (9182.01 mm^3^, *d =* 0.66 [-0.16 to 1.46], *P* = .158). Differences between DTG- and EFV-exposed children were generally small to moderate in magnitude across subcortical regions (Figure 2), however, CIs were wide and statistically significant differences were not detected (Table 2). For example, effect size estimates included the thalamus (adjusted difference -238.98 mm^3^, *d =* -0.32 [-1.12 to 0.47], *P* = .479), caudate (284.71 mm^3^, *d =* 0.32 [-0.48 to 1.10], *P* = .484), putamen (714.46 mm^3^, *d* = 0.51 [-0.30 to 1.3], *P* = .272), pallidum (161.04 mm^3^, *d* = 0.48 [-0.32 to 1.27], *P* = .292), hippocampus (-37.96 mm^3^, *d =* -0.07 [-0.85 to 0.72], *P* = .885), and amygdala (-58.68 mm^3^, *d =* -0.20 [-0.98 to 0.59], *P* = .666) (Table 2). There were also no detectable differences in cortical thickness or surface area between ART exposure (*P* > .1, Tables S2 and S3).

**Table 2:** Adjusted Mean Differences in Global and Regional Gray Matter Volumes between Children According to HIV Exposure (CHEU vs CHU) and ART Exposure (DTG vs EFV) Status

Brain volume (mm^3^)	CHEU mean (SD)	CHU mean (SD)	Adjusted ^a^ difference (95% CI)	*P*-value	Cohen’s d (effect size)	DTG mean (SD)	EFV mean (SD)	Adjusted^b^ difference (95% CI)	*P*-value	Cohen’s d (Effect size)
*Global*										
Total gray matter	671708.64 (52399.09)	671498.51 (61727.25)	1982.61 (−11855.57 to 15820.79)	.775	0.08 (−0.44 to 0.60)	668574.10 (69326.79**)**	675104.39 (26892.74)	−1386.54 (−17689.1 to 14916.02)	.861	−0.08 (−0.86 to 0.71)
Cerebral white matter	345040.68 (29875.13)	346601.48 (47679.93)	3048.24 (−12903.24 to 18999.72)	.703	0.10 (−0.42 to 0.62)	348985.69 (37365.59)	340766.92 (19644.13)	9182.01 (−3884.93 to 22248.95)	.158	0.66 (−0.16 to 1.46)
*Subcortical*										
Thalamus	12496.71 (761.45)	12464.37 (1386.44)	93.09 (−470.04 to 656.2)	.742	0.09 (−0.43 to 0.61)	12393.98 (837.98)	12608.01 (687.76)	−238.98 (−930.95 to 452.99)	.479	−0.32 (−1.12 to 0.47)
Caudate	7117.14 (948.43)	7340.02 (1044.21)	−168.42(−657.99 to 321.16)	.493	−0.19 (−0.71 to 0.34)	7296.18 (1109.74)	6923.12 (734.99)	284.71 (−550.69 to 1120.09)	.484	0.32 (−0.48 to 1.10)
Putamen	9111.72 (1576.99)	9492.98 (1129.32)	−283.68 (−917.04 to 349.68)	.373	−0.24 (−0.76 to 0.28)	9447.34 (1446.21)	8748.14 (1693.21)	714.4637 (−606.60 to 2035.53)	.272	0.51 (−0.30 to 1.30)
Pallidum	3393.42 (435.90)	3515.10 (520.49)	−65.41 (−269.31 to 138.50)	.523	−0.17 (−0.69 to 0.35)	3483.33 (462.22)	3296.03 (401.98)	161.04 (−150.18 to 472.25)	.292	0.48 (−0.32 to 1.27)
Hippocampus	6352.51 (805.88)	6600.43 (842.71)	−189.21 (−511.06 to 132.64)	.244	−0.32 (−0.84 to 0.21)	6352.37 (989.86)	6352.67 (589.99)	−37.96 (−581.77 to 505.85)	.885	−0.07 (−0.85 to 0.72)
Amygdala	2489.11 (495.79)	2543.26 (336.75)	35.31 (−186.29 to 115.67)	.641	−0.13 (−0.65 to 0.39)	2458.76 (626.37)	2521.99 (325.64)	−58.68 (−339.14 to 221.79)	.666	−0.20 (−0.98 to 0.59)

Multiple linear regression estimates for HIV and ART exposure on global and subcortical brain volume.

^a^Adjusted for age, sex, intracranial volume.

^b^Adjusted for age, sex intracranial volume and maternal CD4. Global and subcortical volume (mean total of left and right hemispheres), mean differences (adjusted regression coefficients with 95% CIs in multiple regression models), *P*-values, Cohen’s D effect sizes (95% CIs) are presented here. Abbreviations: CHEU, children who are HIV-exposed uninfected; CHU, children who are HIV-unexposed; CI, confidence interval; DTG, dolutegravir; EFV, efavirenz; SD, standard deviation. ``Brain volume (mm3) region'' may be more appropriate for this column heading, or just ``region'' with the added heading of Brain volume (mm3) over CHEU and CHU columns.

### HIV Exposure and Brain Structure

Total intracranial volume was similar between CHEU and CHU (1286 vs 1296 cm^3^, *P* = .822) (Table 1). Multiple linear regression estimates showed no detectable brain volume differences and small effect size differences between CHEU and CHU in total gray matter (adjusted difference 1982.61 mm^3^, Cohen’s *d =* 0.08 [95% CI -0.44 to 0.60], *P* = .775) and total white matter (3048.24 mm^3^, *d =* -0.10 [-0.44 to 0.60], *P* = .775), or subcortical ROIs including the thalamus (93.09 mm^3^, *d =* 0.09 [-0.43 to 0.61], *P* = .742), caudate (-168.42 mm^3^, *d =* -0.19 [-0.71 to 0.34], *P* = .493), putamen (-283.68 mm^3^, *d =* -0.24 [-0.76 to 0.28], *P* = .373), pallidum (-65.41 mm^3^, *d =* -0.17 [-0.69 to 0.35], *P* = .523), hippocampus (-189.21 mm^3^, *d =* -0.32 [-0.84 to 0.21], *P* = .244), and amygdala (35.31 mm^3^, *d =* -0.13 [-0.65 to 0.39], *P* = .641) (Table 2). Similarly, multiple linear regression estimates showed no association of HIV exposure with brain surface area or cortical thickness (*P* > .05, Tables S2 and S3).

### Maternal HIV Disease Severity and Child Brain Structure

In exploratory analyses, associations between maternal CD4 count during pregnancy and child brain volumes were small and not statistically significant (*P* > .3, Table S4). Similarly, maternal viral load (log-transformed), measured at enrolment in the third trimester prior to ART initiation and at delivery, was not significantly associated with child brain volume outcomes (*P* > .1, Table S4).

## DISCUSSION

Dolutegravir is established as the first-line treatment for HIV in pregnancy, yet data on its long-term impact on child brain structural development remain limited. In this study, we found no detectable differences in brain morphology, including volume, cortical thickness, or surface area, between children with in-utero exposure to DTG versus EFV in late-stage pregnancy, nor between CHEU and CHU. To our knowledge, this is the first study to use MRI to assess brain structure of children with perinatal exposure to DTG. These findings contribute novel neuroimaging data and insights on early brain structure in the context of this more recent treatment.

In this small sample, children with in-utero exposure to DTG- and EFV-based ART initiated in late pregnancy demonstrated no detectable brain structural differences across all measured domains. A key advantage of DTG over EFV in pregnancy is its superior efficacy in achieving maternal viral suppression at the time of birth. The DolPHIN-2 clinical trial demonstrated pregnant women who initiated DTG-based therapy in the third trimester were more likely to achieve viral suppression (<50 ^21^ copies/mL) at delivery.^21^ DTG and EFV differ in their mechanism of action and pharmacokinetic profiles. DTG is an integrase strand transfer inhibitor that prevents viral DNA integration into the host genome, resulting in rapid and sustained viral suppression, whereas EFV is a non-nucleoside reverse transcriptase inhibitor that acts earlier in the viral replication cycle but is associated with greater central nervous system penetration and neuropsychiatric side effects in adults.^21,27^ Despite these pharmacological differences, both regimens resulted in comparable maternal viral suppression at delivery in this neuroimaging sub-study, conducted with a smaller South African subset of participants from DolPHIN-2. This comparable viral suppression in this sub-sample may partly explain the lack of detectable differences in brain structure between DTG- and EFV- exposed children. Early and effective viral suppression of HIV is critical for several general health reasons, such as preventing HIV transmission, but also for reducing the potential neurotoxic effects of the virus and associated maternal immune activation on fetal brain development. Additionally, high levels of alcohol exposure, which were observed across all groups in this study, may have obscured more subtle ART or HIV-related effects on brain structural development. To our knowledge, only one other neuroimaging study has been set up to investigate brain structure in CHEU with a focus on ART exposure. In that study, neonates with prenatal exposure to HIV and EFV-based ART had smaller left putamen and bilateral caudate volumes compared to their HIV-unexposed counterparts.^17^ Importantly, reductions in caudate volume were dependent on the duration of in-utero ART-exposure, with effects only seen in neonates born to mothers initiating ART post-conception.^17^ This suggests that earlier and sustained ART may offer neuroprotection to subcortical brain structures. Although our study could not assess the impact of ART initiation timing, this remains an important consideration in optimizing fetal brain development among CHEU, particularly in the context of expanding DTG use in pregnancy.

We conducted an exploratory analysis within the CHEU group to examine whether maternal HIV disease severity, as measured by viral load and CD4 count, was associated with child brain volume. No significant associations were detected in our cohort. Maternal immune health during pregnancy is thought to play an important role in early development, as previous studies have shown that lower maternal CD4 and higher viral load are associated with an increased risk of infant morbidity and mortality.^28^ Although the exact mechanisms underlying this remain unclear, they are likely multifactorial.^29^ Maternal immune dysregulation has previously been linked to various neurological disorders and abnormal brain development.^30^ It is therefore plausible that chronic exposure to maternal inflammation caused by HIV infection may impact fetal brain development with lasting effects. This hypothesis is supported by findings from the Drakenstein Child Health Study (DCHS) birth cohort, where lower maternal CD4 count was found to be associated with smaller gray matter and putamen volumes in CHEU as neonates and 2-3 years of age, respectively.^18,19,31^ While both the DCHS and DolPHIN-2 PLUS studies were conducted in similar settings in South Africa, the DolPHIN-2 PLUS children represent a more recent cohort of children born between 2018 and 2019. Global and national efforts to expand ART coverage and strengthen prenatal care in mothers living with HIV in South Africa may be contributing to improved maternal health and pregnancy outcomes, and consequently, healthier children.^32,33^ Although our analyses were limited by small sample size, it is possible the lack of association between maternal CD4 and child brain volume reflects better overall prenatal health and HIV management in this more recent cohort. Further research is needed to determine if this is evident in other current birth cohorts.

When comparing CHEU and CHU groups among our small sample, we did not detect associations between HIV exposure and brain volume, surface area, or thickness. This contrasts previous studies of infants and young children, which have reported altered structural brain development in CHEU, however none have examined children with DTG exposure.^17–19^ Recent MRI research conducted in South Africa has reported reduced volumes in gray matter, and basal ganglia regions including the caudate and left putamen, in CHEU when compared to their unexposed counterparts, although changes in later childhood are less consistent.^17,18,20,34^ In addition to altered brain volume, increased cortical thickness in prefrontal regions such as the medial orbitofrontal cortex has been found in CHEU at 2-3 years of age.^35^ The similar brain structure observed between CHEU and CHU in this study may have been driven by the use of newer and more effective HIV treatments for use in late pregnancy, which may be protecting the developing brain from the HIV-related effects seen in older studies. Additionally, mothers in the CHEU group were part of the DolPHIN-2 clinical trial, and as a result, may have had better access to perinatal and follow-up care and support, which could have mitigated the impact on brain structural development associated with exposure to HIV and ART in early life. This study had a smaller sample size compared to DCHS and, therefore, may have been underpowered to detect the subtle effects of HIV exposure on the developing brain.

Although not statistically significant, a higher proportion of mothers of CHU reported depressive symptoms compared to mothers of CHEU (18% vs 8%), an unexpected pattern that may reflect unmeasured psychosocial or environmental stressors in the CHU group. Maternal depression has been linked to altered neurodevelopment and may have influenced brain outcomes independent of HIV or ART exposure.^36^ It is also possible that other unmeasured confounders, such as maternal health, quality of prenatal care, and environmental exposures present in the CHU group, may have influenced brain development and contributed to the observed lack of differences between CHEU and CHU.

A key strength of this study was the availability of detailed maternal HIV health metrics gathered in mothers of CHEU in late pregnancy, which allowed us an in-depth analysis of maternal HIV and ART and brain structure in CHEU. However, the timing of maternal HIV diagnosis and infection prior to trial enrolment was not recorded or available, limiting our ability to determine ART duration or whether mothers in the CHEU group had chronic HIV infection or were recently seroconverted. Consequently, immune status prior to the third trimester could not be assessed. The pregnancy health records of mothers of CHU were not available, as this group was recruited postnatally, thus limiting our ability to assess other important factors related to maternal immune status that may impact brain development regardless of HIV/ART exposure.

Environment exposure may also have influence brain outcomes. High levels of maternal alcohol exposure were reported across all study groups, which may have obscured more subtle ART- or HIV-related effects on brain structural development.

Additional limitations inherent to pediatric MRI studies may result from scanning young children in non-sedated sleep, which can introduce motion artifacts and lead to data exclusion despite careful quality control procedures.

Finally, the relatively small proportion of children from the original South African DolPHIN-2 cohort who participated in the DolPHIN-2 PLUS neuroimaging follow-up, raising the possibility of selection bias. Attrition occurred over the prolonged interval between the parent trial and the imaging sub-study and was exacerbated by COVID-19−related disruptions to in-person research activities. Disruptions included difficulties re-establishing contact with families, relocation outside the study catchment area, and children falling outside the predefined age window at the time of contact. Although comparisons with the broader South African DolPHIN-2 cohort demonstrated similar maternal sociodemographic and HIV-related characteristics between cohorts, including maternal education, employment status, CD4 count, and viral load, differences in maternal depressive symptoms observed between ART groups in the parent trial were not detected in the neuroimaging sub-sample. This likely reflects limited statistical power rather than systematic selection based on psychosocial factors. Overall, comparisons with the parent South African DolPHIN-2 cohort suggest that, despite substantial attrition, the DolPHIN-2 PLUS neuroimaging sub-sample was broadly representative with respect to key maternal sociodemographic and HIV-related characteristics. However, unmeasured characteristics associated with follow-up participation may remain and could limit the generalizability of findings. The small sample size, particularly within the DTG and EFV comparison groups, reduces the statistical power and generalizability of these findings, and smaller differences in brain structure may therefore have gone undetected.

We acknowledge there may have been other unmeasured factors that could have impacted our findings. Nonetheless, these findings contribute valuable insight into the relationship between maternal ART and brain structural development. Future longitudinal research is needed to investigate the developmental trajectory of children exposed to DTG and other newer ART, the effects of ART timing and duration, and the relationship between maternal immune health and brain structure. Diffusion tensor imaging, which provides information on white matter connectivity and brain microstructure, was not included in this study and represents an important avenue for future research examining brain network development in CHEU. Larger sample sizes assessing both structural and functional outcomes will further clarify the long-term impact of DTG on child neurodevelopment.

## CONCLUSION

In this cohort of children aged 3-4 years, no differences in brain structure were detected between those exposed to DTG or EFV in late pregnancy, or between CHEU and CHU. Although several regions demonstrated small-to-moderate effect size estimates, this small sample size was under powered to detect statistically significant differences in brain structure. More research with larger sample sizes is needed to replicate these findings and elucidate any protective effect that earlier ART initiation may offer. Our findings provisionally support the use of DTG in late pregnancy with respect to early childhood brain structural outcomes. Ongoing monitoring and further research are crucial to fully clarify the effects of prenatal exposure to antiretroviral therapies on long-term neurodevelopment. These efforts are essential for optimizing treatment guidelines and improving outcomes for children born to mothers living with HIV.

## Supplementary Material

piag022_Supplementary_Tables

## Data Availability

The data that support the findings of this study are not publicly available due to participant confidentiality requirements, but may be available from the corresponding author on reasonable request.
