# Supplementary material for: Brain Structure of South African Children Born to Mothers on Dolutegravir Versus Efavirenz-Based Antiretroviral Therapy
Source: J Pediatric Infect Dis Soc. 2026 Mar 27;15(5):piag022. doi: 10.1093/jpids/piag022 (PMC13221953; doi:10.1093/jpids/piag022)
Supplement: piag022_Supplementary_Tables [file piag022_supplementary_tables.docx]

**Supplementary Table S1**. Socio-demographic and clinical characteristics of pregnant mothers enrolled in DolPHIN-2 trial in South Africa

| **Variable** | **Total**  **(N = 129)** | **DTG**  **(N = 65)** | **EFV**  **(N = 64)** | **p-value** |
| --- | --- | --- | --- | --- |
| *Sociodemographic characteristics* | | | | |
| Maternal Education (Completed secondary school)^a^ | 42 (45%) | 20 (40%) | 22 (50%) | 0.331 |
| Maternal Employment Status (Employed)^a^ | 29 (32%) | 17 (35%) | 12 (28%) | 0.443 |
| Maternal Depression | 14 (11%) | 11 (17%) | 3 (5%) | 0.025^*^ |
| *Maternal HIV variables in pregnancy* | | | | |
| Maternal CD4 count, median (IQR) (cells/mm^3^)^b^ | 360 (263 - 541) | 410 (248 – 660) | 355 (266 – 478) | 0.140 |
| ≤500 cells/mm^3^ | 87 (69%) | 39 (61%) | 48 (76%) | 0.064 |
| >500 cells/mm^3^ | 40 (31%) | 25 (39%) | 15 (24%) |  |
| Maternal Viral Load at enrolment (copies/ml) |  |  |  |  |
| <50 copies/ml | 6 (5%) | 3 (5%) | 3 (5%) | 0.984 |
| ≥50 copies/ml | 123 (95%) | 62 (95%) | 61 (95%) |  |
| Maternal Viral Load at delivery (copies/ml)^c^ |  |  |  |  |
| <50 copies/ml | 74 (64%) | 47 (81%) | 27 (47%) | <0.001^*^ |
| ≥50 copies/ml | 41 (36%) | 11 (19%) | 30 (53%) |  |

Data are N (%), mean (SD), or median (IQR). Continuous variables were compared with unpaired t-tests if normally distributed or Mann-Whitney U tests if distribution was not normal; categorical variables were compared with chi-squared tests. *p<0.05. ^a^N=35 (N=15 DTG; N=20 EFV) missing data for measurements of maternal education and employment status. ^b^N=2 (N=1 DTG; N=1 EFV) missing data for measurement of maternal CD4 count at enrolment. ^c^N=14 (N=7 DTG; N=7 EFV) missing data for measurement of maternal viral load at delivery. *Abbreviations: DTG, dolutegravir; EFV, efavirenz*

**Alt text:** Table summarizing maternal demographic and clinical characteristics of pregnant mothers enrolled in DolPHIN-2 trial by ART exposure (DTG vs EFV). Variables include child age, sex, maternal education and employment, substance use, depression, intracranial volume, CD4 count and viral load.

**Supplementary Table S2.** Adjusted mean differences of brain surface areas between children according to HIV exposure (CHEU vs CHU) and ART exposure (DTG vs. EFV) status

| **Total Surface Area** | **CHEU mean (SD)** | **CHU mean (SD)** | **Adjusted^a^ difference** | **P-value** | **Cohen’s d (effect size)** | **DTG**  **mean (SD)** | **EFV**  **mean (SD)** | **Adjusted^b^ difference** | **P-value** | **Cohen’s d (effect size)** |
| --- | --- | --- | --- | --- | --- | --- | --- | --- | --- | --- |
| Frontal Lobe | 55607.56 (5343.82) | 55898.91 (6059.79) | 83.84 (-1995.61 to 2163.3) | 0.936 | 0.02 (-0.50 to 0.54) | 55528.62 (7086.20) | 55693.08 (2740.52) | 480.35 (-2522.23 to 3482.93) | 0.741 | 0.15 (-0.64 to 0.93) |
| Temporal Lobe | 29873.16 (2880.82) | 29972.03 (3378.98) | 84.42 (-1006.27 to 1175.12) | 0.877 | 0.04 (-0.48 to 0.56) | 30065.69 (3871.58) | 29664.58 (1290.02) | 580.22 (-917.13 to 2077.56) | 0.427 | 0.36 (-0.43 to 1.12) |
| Parietal Lobe | 44070.68 (4236.88) | 44114.15 (5804.56) | 359.00 (-1883.95 to 2601.5) | 0.749 | 0.09 (-0.43 to 0.61) | 44665.23 (5117.48) | 43426.58 (3118.72) | 830.43 (-2429.05 to 4089.92) | 0.600 | 0.24 (-0.55 to 1.022) |
| Occipital Lobe | 22500.32 (3294.56) | 22089.39 (2786.22) | 341.89 (-1109.17 to 1792.94) | 0.638 | 0.13 (-0.39 to 0.65) | 21674.62 (3578.02) | 23394.83 (2834.96) | -1384.77 (-3659.57 to 890.02) | 0.218 | -0.57 (-1.36 to 0.24) |
| Cingulate Cortex | 6757.32 (692.92) | 6765.67 (898.98) | 39.97 (-294.94 to 374.88) | 0.812 | 0.06 (-0.46 to 0.58) | 6839.08 (883.10) | 6668.75 (424.69) | 166.01 (-360.32 to 692.34) | 0.517 | 0.29 (-0.50 to 1.08) |
| Insular Cortex | 4217.88 (393.99) | 4108.46 (495.74) | 111.76 (-120.30 to 343.82) | 0.338 | 0.26 (-0.26 to 0.78) | 4240.39 (381.40) | 4193.50 (422.78) | 97.26 (-244.00 to 438.51) | 0.558 | 0.27 (-0.53 to 1.05) |

Multiple linear regression estimates for HIV and ART exposure on brain surface area.^a^ Adjusted for age, sex, intracranial volume. ^b^Adjusted for age, sex intracranial volume and maternal CD4. Total surface area (total mean of left and right hemispheres combined), mean differences (adjusted regression coefficients with 95% confidence intervals in multiple regression models), p-values are presented here. *Abbreviations: CHEU, Children who are HIV-exposed uninfected; CHU, children who are HIV-unexposed; DTG, dolutegravir; EFV, efavirenz; CI, confidence interval; SD, standard deviation.*

**Alt text**: Table displaying adjusted mean differences in cortical surface area across six brain lobes (frontal, temporal, parietal, occipital, cingulate, insular) by HIV and ART exposure status. No significant differences observed.

**Supplementary Table S3.** Adjusted mean differences of cortical thickness between children according to HIV exposure (CHEU vs CHU) and ART exposure (DTG vs. EFV)

| **Mean Cortical Thickness** | **CHEU**  **mean (SD)** | **CHU**  **mean (SD)** | **Adjusted^a^ difference** | **P-value** | **Cohen’s d**  **(effect size)** | **DTG**  **mean (SD)** | **EFV**  **mean (SD)** | **Adjusted^b^ difference** | **P-value** | | **Cohen’s d (effect size)** |
| --- | --- | --- | --- | --- | --- | --- | --- | --- | --- | --- | --- |
| Frontal Lobe | 2.89 (0.11) | 2.91 (0.14) | -0.03 (-0.10 to 0.04) | 0.406 | -0.23 (-0.75 to 0.30) | 2.90 (0.13) | 2.88  (0.10) | 0.02 (-0.09 to 0.12) | 0.776 | 0.13 (-0.66 to 0.91) | |
| Temporal Lobe | 2.95 (0.10) | 2.94 (0.14) | -0.01 (-0.08 to 0.06) | 0.750 | -0.09 (-0.61 to 0.43) | 2.94 (0.09) | 2.96  (0.11) | -0.02 (-0.12 to 0.08) | 0.656 | -0.20 (-0.98 to 0.59) | |
| Parietal Lobe | 2.70 (0.08) | 2.69 (0.11) | 0.01 (-0.04 to 0.06) | 0.735 | 0.01 (-0.04 to 0.06) | 2.71 (0.09) | 2.70  (0.08) | 0.02 (-0.05 to 0.10) | 0.538 | 0.28 (-0.52 to 1.06) | |
| Occipital Lobe | 2.24 (0.10) | 2.24 (0.11) | 0.01 (-0.05 to 0.06) | 0.861 | 0.05 (-0.47 to 0.57) | 2.27 (0.10) | 2.22  (0.10) | 0.07 (-0.02 to 0.17) | 0.112 | 0.73 (-0.09 to 1.54) | |
| Cingulate Cortex | 2.71 (0.14) | 2.69 (0.15) | 0.00 (-0.07 to 0.07) | 0.977 | -0.01 (-0.53 to 0.51) | 2.70 (0.15) | 2.73  (0.14) | -0.04 (-0.16 to 0.09) | 0.575 | -0.25 (-1.04 to 0.54) | |
| Insular Cortex | 3.14 (0.15) | 3.21 (0.19) | -0.09 (-0.18 to 0.00) | 0.052 | -0.54 (-1.06 to -0.01) | 3.13 (0.12) | 3.16  (0.18) | -0.06 (-0.20 to 0.09) | 0.412 | -0.37 (-1.16 to 0.43) | |

Multiple linear regression estimates for HIV and ART exposure on brain cortical thickness..^a^ Adjusted for age and sex,. ^b^Adjusted for age, sex and maternal CD4. Cortical thickness (mean of left and right hemispheres), mean differences (adjusted regression coefficients with 95% confidence intervals in multiple regression models), p-values are presented here. *Abbreviations: CHEU, Children who are HIV-exposed uninfected; CHU, children who are HIV-unexposed; DTG, dolutegravir; EFV, efavirenz; CI, confidence interval; SD, standard deviation.*

**Alt text**: Table displaying adjusted mean differences in cortical surface area across six brain lobes (frontal, temporal, parietal, occipital, cingulate, insular) by HIV and ART exposure status. No significant differences observed.

**Supplementary Table S4.** Adjusted mean differences in global and regional grey matter volumes between children according to maternal CD4 count in pregnancy

| **Maternal CD4 count at enrolment (cells/mm3)** | **Mean (SD)** | **Adjusted^a^ coefficient (95% CI)** | **P-value** |
| --- | --- | --- | --- |
| *Global* |  |  |  |
| **Total Grey**  CHU  CD4 >500 cells/mm^3^  CD4 ≤500 cells/mm^3^ | 671498.50 (61727.25)  674992.20 (55579.73)  669519.60 (52040.02) | Reference  -3888.47 (-22476.46 to 14699.53)  6181.67 (-10266.23 to 22629.56) | 0.613 |
| **Cerebral White matter**  CHU  CD4 >500 cells/mm^3^  CD4 ≤500 cells/mm^3^ | 346601.50 (47679.93)  353921.30 (34011.71)  339120.30 (26315.35) | Reference  4589.18 (-17013.39 to 26191.75)  1946.14 (-17169.25 to 21061.53) | 0.909 |
| *Subcortical* |  |  |  |
| **Thalamus**  CHU  CD4 >500 cells/mm^3^  CD4 ≤500 cells/mm^3^ | 12464.37 (1386.44)  12461.77 (912.08)  12520.01 (676.55) | Reference  -76.46 (-836.13 to 683.20)  214.35 (-457.85 to 886.55) | 0.757 |
| **Caudate**  CHU  CD4 >500 cells/mm^3^  CD4 ≤500 cells/mm^3^ | 7340.02 (1044.21)  7278.99 (1091.83)  7009.25 (862.85) | Reference  -117.44 (-780.41 to 545.53)  -204.88 (-791.52 to 381.76) | 0.772 |
| **Putamen**  CHU  CD4 >500 cells/mm^3^  CD4 ≤500 cells/mm^3^ | 9492.98 (1129.32)  9531.81 (1211.55)  8831.67 (1763.39) | Reference  -41.76 (-893.91 to 810.39)  -456.70 (-1210.74 to 297.34) | 0.470 |
| **Pallidum**  CHU  CD4 >500 cells/mm^3^  CD4 ≤500 cells/mm^3^ | 3515.10 (520.49)  3599.47 (366.87)  3256.06 (434.35) | Reference  53.95 (-217.78 to 325.68)  -150.78 (-391.22 to 89.67) | 0.346 |
| **Hippocampus**  CHU  CD4 >500 cells/mm^3^  CD4 ≤500 cells/mm^3^ | 6600.43 (842.71)  6525.16 (1008.88)  6237.41 (650.79) | Reference  -139.60 (-575.18 to 295.97)  -224.69 (-610.11 to 160.74) | 0.481 |
| **Amygdala**  CHU  CD4 >500 cells/mm^3^  CD4 ≤500 cells/mm^3^ | 2543.26 (336.75)  2554.92 (466.18)  2445.24 (525.85) | Reference  -21.01 (-225.48 to 183.46)  -45.53 (-226.46 to 135.40) | 0.878 |

Multiple linear regression estimates for maternal CD4 and brain volume.^a^ Adjusted for age, sex and intracranial volume. Global and subcortical volume (mean total of left and right hemispheres) dichotomized by maternal CD4 count, mean differences (adjusted regression coefficients with 95% confidence intervals in multiple regression models), overall p-values are presented here. *Abbreviations: CHU, children who are HIV-unexposed; CI, confidence interval; SD, standard deviation.*

**Alt text:** Table presenting regression estimates of child brain volumes (global and subcortical) by maternal CD4 count during pregnancy. No statistically significant associations found between maternal immune status and brain metrics.
